# Alpha-Synuclein Oligomers Interact with Metal Ions to Induce Oxidative Stress and Neuronal Death in Parkinson's Disease

**DOI:** 10.1089/ars.2015.6343

**Published:** 2016-03-01

**Authors:** Emma Deas, Nunilo Cremades, Plamena R. Angelova, Marthe H.R. Ludtmann, Zhi Yao, Serene Chen, Mathew H. Horrocks, Blerida Banushi, Daniel Little, Michael J. Devine, Paul Gissen, David Klenerman, Christopher M. Dobson, Nicholas W. Wood, Sonia Gandhi, Andrey Y. Abramov

**Affiliations:** ^1^ Department of Molecular Neuroscience, UCL Institute of Neurology, Queen Square, London, United Kingdom.; ^2^ Department of Chemistry, Lensfield Road, University of Cambridge, Cambridge, United Kingdom.; ^3^ Sobell Department of Motor Neuroscience and Movement Disorders, UCL Institute of Neurology, Queen Square, London, United Kingdom.; ^4^ MRC Laboratory for Molecular Cell Biology, UCL, London, United Kingdom.

## Abstract

***Aims:*** Protein aggregation and oxidative stress are both key pathogenic processes in Parkinson's disease, although the mechanism by which misfolded proteins induce oxidative stress and neuronal death remains unknown. In this study, we describe how aggregation of alpha-synuclein (α-S) from its monomeric form to its soluble oligomeric state results in aberrant free radical production and neuronal toxicity. ***Results:*** We first demonstrate excessive free radical production in a human induced pluripotent stem-derived α-S triplication model at basal levels and on application of picomolar doses of β-sheet-rich α-S oligomers. We probed the effects of different structural species of α-S in wild-type rat neuronal cultures and show that both oligomeric and fibrillar forms of α-S are capable of generating free radical production, but that only the oligomeric form results in reduction of endogenous glutathione and subsequent neuronal toxicity. We dissected the mechanism of oligomer-induced free radical production and found that it was interestingly independent of several known cellular enzymatic sources. ***Innovation:*** The oligomer-induced reactive oxygen species (ROS) production was entirely dependent on the presence of free metal ions as addition of metal chelators was able to block oligomer-induced ROS production and prevent oligomer-induced neuronal death. ***Conclusion:*** Our findings further support the causative role of soluble amyloid oligomers in triggering neurodegeneration and shed light into the mechanisms by which these species cause neuronal damage, which, we show here, can be amenable to modulation through the use of metal chelation. *Antioxid. Redox Signal*. 24, 376–391.

## Introduction

Neurodegenerative diseases are becoming increasingly prevalent in the aging populations of the Western world. These disorders, which include Parkinson's disease (PD), share a common feature of the accumulation of abnormally aggregated proteins in pathological inclusions containing amyloid fibrils (13, 29). While the protein that is deposited varies from one disease to another, the formation of the inclusions occurs by a generic process of misfolding, by which a normally soluble protein converts into fibrillar aggregates *via* a series of oligomeric intermediates and ultimately insoluble fibrils that are deposited in the brain. Accumulating evidence suggests that soluble oligomeric species generated during the formation of fibrils are the most neurotoxic species linked with the development of these types of diseases (8, 35, 38, 49, 53).

PD is characterized by the loss of midbrain dopaminergic neurons and the presence of alpha-synuclein (α-S) neuronal aggregated inclusions, known as Lewy bodies and Lewy neurites. Rare forms of autosomal dominant familial PD can be attributed solely to mutations in the SNCA gene or by genetic duplication or triplication of the wild-type *SNCA* locus (46). Duplication or triplication of the *SNCA* gene correlates with a younger age of disease onset and severity, suggesting that there is a dose-dependent effect of the protein in disease causation. These genetic and pathological data suggest that dysfunction/misfolding of the α-S protein is a primary step in disease pathogenesis and is sufficient to trigger the development of PD. However, the underlying mechanism by which α-S aggregation induces neuronal death during disease remains unknown.

Innovation Our findings indicate that certain structural groups of soluble oligomeric species formed during alpha-synuclein amyloid fibril formation are especially damaging to healthy primary neuronal cells and human induced pluripotent stem cell-derived neurons through the induction of aberrant production of cytosolic ROS in a metal ion-dependent manner that ultimately results in cell toxicity. We propose that this linked process between amyloid aggregation, induction of oxidative stress, and neuronal death is likely central in the pathogenesis of Parkinson's disease.

Strong evidence exists to support a role of oxidative stress in the pathogenesis of many neurodegenerative diseases, including PD. There is clear evidence of oxidative damage to lipids, proteins, and DNA (22) in postmortem PD brain. Basal lipid peroxidation in substantia nigra is increased in PD, leading to damage of intracellular components and apoptotic cell death, both of which have been detected in autopsy tissue from the brains of individuals with PD (43, 54). Animal models of PD based on toxins (MPTP, rotenone, paraquat, and 6-OHDA) induce oxidative stress and dopaminergic cell death and recapitulate several of the motor and pathological aspects of PD.

Several of the genes known to cause familial PD also impact on mitochondrial dysfunction and the generation of reactive oxygen species (ROS) and susceptibility to oxidative stress, including *PINK1*, *parkin*, *DJ-1*, *SNCA*, and *LRRK2* (18, 24, 28). While a number of different processes are recognized to generate ROS in sporadic PD, namely mitochondrial dysfunction, dopamine metabolism, and iron and calcium homeostasis, there remains a fundamental gap in our understanding of how protein aggregation of α-S can impact on the generation of ROS.

In this study, our aim has been to (i) study the role of ROS production in a novel induced pluripotent stem cell (iPSC)-derived neuronal model of PD bearing *SNCA* triplication, (ii) identify which structural form of α-S is responsible for ROS production, (iii) determine the mechanisms by which α-S induces ROS generation, and (iv) investigate the relevance of α-S-induced ROS production in disease. To address these objectives, we have utilized iPSC neurons derived from an *SNCA* triplication patient (20) to assess the effects of long-term exposure to increased levels of intracellular α-S and also two types of exogenously produced highly characterized monomeric, oligomeric, and fibrillar forms of α-S to identify the conformational state of the protein primarily responsible for toxicity.

## Results

### Human iPSC-derived neurons with *SNCA* triplication have high basal levels of ROS production and oligomer-induced ROS production

**FIG. 1.**
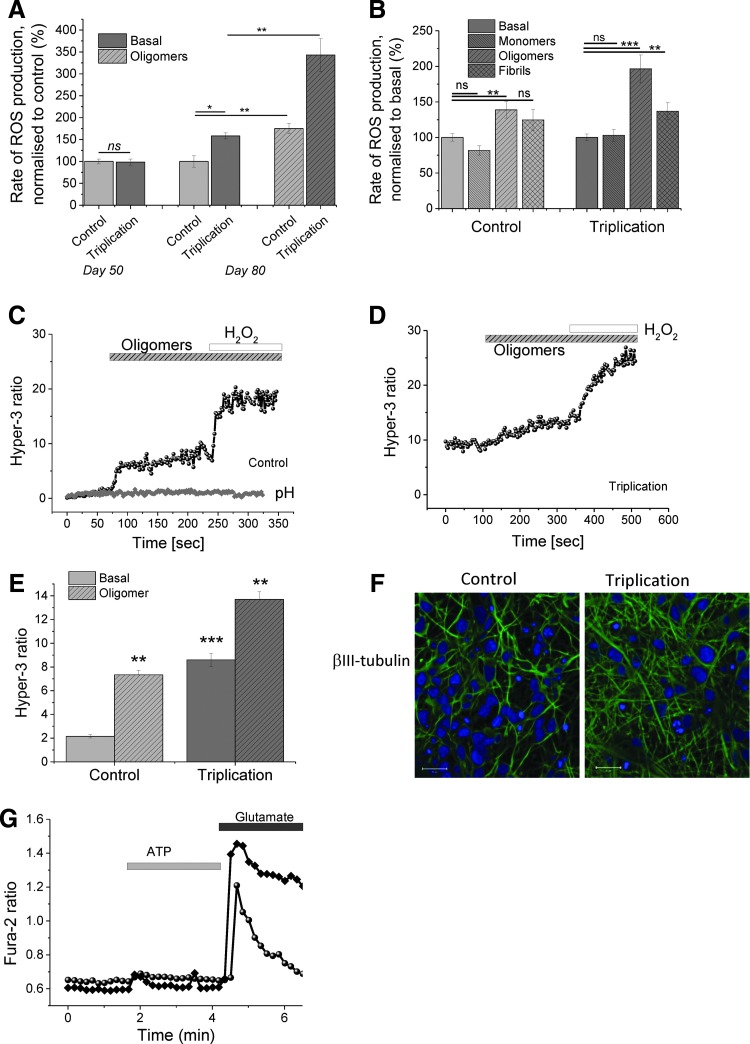
**Oligomeric α-S induces ROS production in control and *SNCA* triplication neurons. (A)** Basal and oligomeric induced levels of ROS were assessed in young (50 days) and old (80 days) control and *SNCA* triplication differentiated neurons using dihydroethidium. Old neurons were exposed to oligomers and the rate of ROS production was recorded. **(B)** Young control and triplication neurons were exposed to 300 n*M* monomers, 300 n*M* oligomers (3 n*M* total oligomers), or 300 n*M* fibrils, and ROS production was recorded and compared with basal ROS production levels. **(C)** Aged neurons (80 days) were transfected with the cellular Hyper-3 construct, and H_2_O_2_ production in the presence of oligomers was recorded. Representative trace of H_2_O_2_ production rate in control aged neurons (*black trace*). A control pH construct (Hyper-CS) reported no effect on intracellular pH (*gray trace*). **(D)** Representative trace of H_2_O_2_ production in triplication neurons exposed to oligomers. **(E)** Quantification of basal and oligomer-induced H_2_O_2_ production in control and triplication neurons. **(F)** Immunocytochemistry for iPSC derived neurons for βIII-tubulin (*green*) and Hoechst (*blue*). **(G)** Cytosolic calcium response to glutamate and ATP in iPSC derived neurons (representative traces). *Bars* indicate the incubation period of either oligomers or H_2_O_2_. *n* ≥ 4 experiments. Error bars indicate SEM; *p < 0.05; **p < 0.01; ***p < 0.001. α-S, alpha-synuclein (protein); H_2_O_2_, hydrogen peroxide; ROS, reactive oxygen species; *SNCA* alpha-synuclein (gene). To see this illustration in color, the reader is referred to the web version of this article at www.liebertpub.com/ars

To investigate the effects of long-term intracellular α-S exposure, we initially assessed ROS production in iPSC-derived neurons generated from an *SNCA* triplication patient (two independent clones) and an unaffected first-degree relative as a control (two independent clones) (20). We employed a standard differentiation protocol to generate iPSC-derived cortical neurons and confirmed the presence of neurons using immunocytochemistry to the neuronal marker βIII-tubulin (Fig. 1F). We further tested their functionality as neurons by testing their calcium response to physiological stimuli, ATP (which elicits a P2Y receptor-mediated calcium response in astrocytes), and glutamate (which elicits a glutamate receptor-mediated voltage-gated calcium channel calcium signal in neurons) (representative traces of neuronal response to glutamate are shown in Fig. 1G). Using this protocol, we generate iPSC-derived neurons, of which 60–70% display a calcium response to glutamate.

We utilized two independent ROS assessment methods: first, we measured superoxide radical production as the rate of increase in the ratio of dihydroethidium (HEt) fluorescence between its oxidized and nonoxidized forms. In this method, we measure the rate at which the DHE dye is oxidized (*i.e*., the slope of the increase in the fluorescence intensity of the oxidized dye), which is proportional to the rate of superoxide generation (30). Second, we assessed the rate of hydrogen peroxide (H_2_O_2_) production using the Hyper-3 genetic quantitative probe (5).

We assessed the basal rate of ROS production at day 50 in culture (termed young neurons) and at day 80 in culture (termed aged neurons). Using both methods, we observed a significantly higher basal level of free radical production in the aged triplication neurons compared with control at day 80 in culture (normalized rate of superoxide production 158.3 ± 6.95 in *SNCA* triplication compared with control of 100%; *n* = 4 independent experiments across two clones, *n* = 92 cells for control, *n* = 62 cells for triplication, *p* = 0.01; Fig. 1A), the basal level of H_2_O_2_ was also significantly higher (8.6 ± 0.56 Hyper-3 ratio in *SNCA* triplication, *n* = 4 experiments compared with 2.15 ± 0.14, *n* = 4; in control; *p* < 0.001; Fig. 1C–E). Importantly, young neurons at day 50 postdifferentiation did not show any difference in basal ROS production between control and *SNCA* triplication (*n* = 3 experiments, 98% ± 7.1% of control, Fig. 1A).

To determine if exposure to exogenous α-S has the capacity to increase the basal ROS rate, we added oligomeric α-S to the cells generated using the method described in (14). Using this method of aggregation, an oligomeric mixture can be generated following 29 h of aggregation, at which time point 0.8–1% of the mixture is oligomeric with a stable compact beta-sheet structure, the rest of the preparation being monomeric (referred to as oligomeric species). Exposure of the aged control and triplication iPSC-derived neurons to 10 n*M* oligomeric species resulted in a significant increase in free radical production in both assays. The rate of DHE fluorescence ratio increased in control cells to 175% ± 11.9% (*n* = 4 experiments, *p* = 0.0062; rate of DHE fluorescence ratio increased in *SNCA* triplication cells to 343% ± 37.2%, *n* = 4 experiments, *p* = 6.0E-05; Fig. 1A).

We utilized a second assay that is able to detect H_2_O_2_ through changes in the fluorescence of the genetic construct Hyper-3. Following application of oligomers, the Hyper-3 ratio increased from a basal value of 2.15 ± 0.14 for control cells to 7.34 ± 0.39 and from a basal value of 8.6 ± 0.56 for triplication cells to 13.7 ± 0.64 (Fig. 1C, E). Of note, the basal level of ROS production was higher in the triplication neurons (d80) compared with control, in keeping with the DHE superoxide assay (Fig. 1D, E). Importantly, we performed parallel experiments with a control Hyper construct (Hyper-CS), which demonstrates altered fluorescence in response to changes in pH. The negative results obtained using the control Hyper construct (Hyper-CS) indicate that these findings are not a result of pH alterations (Fig. 1C, gray line). Addition of H_2_O_2_ at the end of the experiment was used as a positive control for the fluorescence signal from Hyper-3.

To determine whether the effect of the oligomeric α-S species on human neurons was specifically due to the oligomeric species, we applied monomeric (300 n*M*), oligomeric (300 n*M* monomeric, 3 n*M* oligomeric), and fibrillar (300 n*M*) α-S to the young day 50 control and triplication neurons (Fig. 1B) and measured the increase in superoxide production (the DHE fluorescence ratio). There was no significant ROS induction by monomers (control+monomers 81% ± 6.4%, *p* = 0.065, triplication 103% ± 8.4%, *p* = 0.773). Oligomers induced ROS production (control 138% ± 11.36%, *p* = 0.0018; triplication 196% ± 19.5%, *p* = 2.22183E-10). Fibrils induced no significant ROS in control (124% ± 14.5%, *p* = 0.065), but induced ROS in triplication (136% ± 11.8%, *p* = 0.002, Fig. 1B).

Thus, α-S overexpression leads to an increase in basal ROS production in human neurons when cultured for long periods of time. Importantly, aggregated α-S (particularly in its oligomeric form) is able to generate further ROS production in neurons derived from iPSCs in both control and *SNCA* triplication cells.

### The ability of α-S to induce ROS is dependent on its conformational state

In previous studies (14), we reported that exposure of rat midbrain cultures to an aggregated solution of fluorescently labeled α-S containing a specific structural group of β-sheet-rich oligomeric species can elicit a significant increase in cellular ROS production. In the present work, we carried out analogous experiments utilizing a newly described method of generating and purifying α-S oligomers (11). Detailed biophysical characterization of this method has confirmed that it is able to generate a 90% pure oligomer preparation whose structures have ∼30–40% beta-sheet content and whose size varies from 10 to 40 protein molecules. They resemble the most highly compact and stable species that are found to accumulate in α-S aggregation methods characterized by single-molecule fluorescence studies also used in this study. This method generated unlabeled populations of monomeric, β-sheet oligomeric (hereafter referred to as enriched oligomeric), or fibrillar α-S species.

**FIG. 2.**
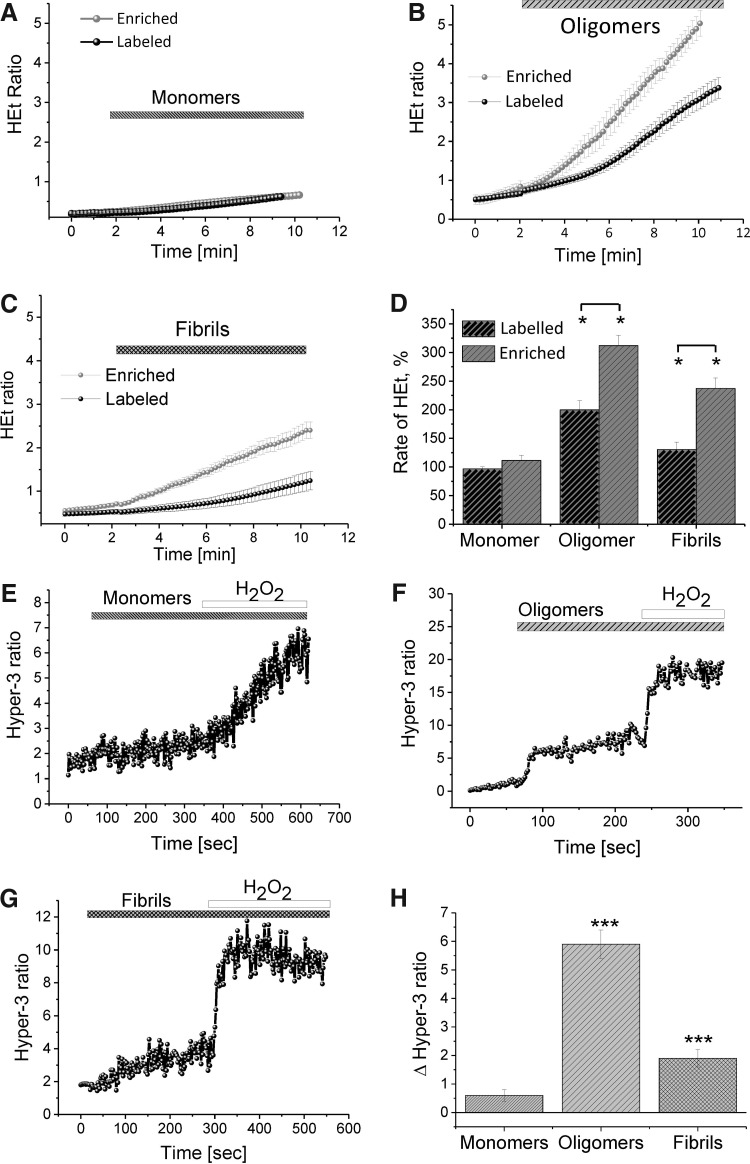
**The ability of α-S to induce ROS is dependent on its structural conformation.** The ability of each different α-S conformation to induce ROS production in WT rat neurons was assessed using both labeled and unlabeled preparations of monomers **(A)**, oligomers **(B)**, and fibrils **(C, D)** Quantification of ROS production in WT rat midbrain neurons upon exposure to α-S species. WT rat midbrain neurons were transfected with the cellular Hyper-3 construct and H_2_O_2_ production in the presence of monomers **(E)**, oligomers **(F)**, and fibrils **(G)** and recorded (representative traces). **(H)** Quantification of α-S-induced H_2_O_2_ production rate in WT rat neurons. *Bars* indicate the incubation period of either α-S species or H_2_O_2_. *n* = 3 experiments. Error bars indicate SEM; *p < 0.05; ***p < 0.001. α-S, α-synuclein; WT, wild-type.

We observed that exposure of the neuronal cultures to monomeric, oligomeric, or fibrillar α-S each produced a different ROS response in the DHE assay (Fig. 2A–D). Specifically, monomeric α-S produced a negligible change in the rate of HEt ratio (with maximal values at 111.4% ± 91% of the basal rate for the addition of 40 n*M*; *n* = 94 cells; Fig. 2A). The enriched oligomers, however, produced a substantially significant increase in the rate of ROS formation with respect to the monomers, indicating that they have a profound impact on cellular ROS production (*n* = 46 cells; 312 ± 18 of basal; *p* < 0.001; Fig. 2B). Fibrils were able to elicit an ROS response in neuronal cells, but this response is significantly lower than that observed after exposure to the oligomeric species (237.4% ± 18% of basal rate; *n* = 32; *p* < 0.005; Fig. 2C).

Comparable results were also obtained when analogous experiments were carried out using the Hyper-3 probe for H_2_O_2_ production (Fig. 2E–H). Specifically, exposure of cells to monomeric α-S showed no significant increase in free radical production (Fig. 2E, H; *n* = 18 cells; *p* > 0.05), while exposure to both the oligomeric (by 5.9 ± 0.5 ratio; *n* = 19 cells; *p* < 0.0001, Fig. 2F, H) and fibrillar species (by 1.9 ± 0.3 ratio; Fig. 2G, H; *n* = 16 cells; *p* < 0.001) was able to induce aberrant H_2_O_2_ production.

**FIG. 3.**
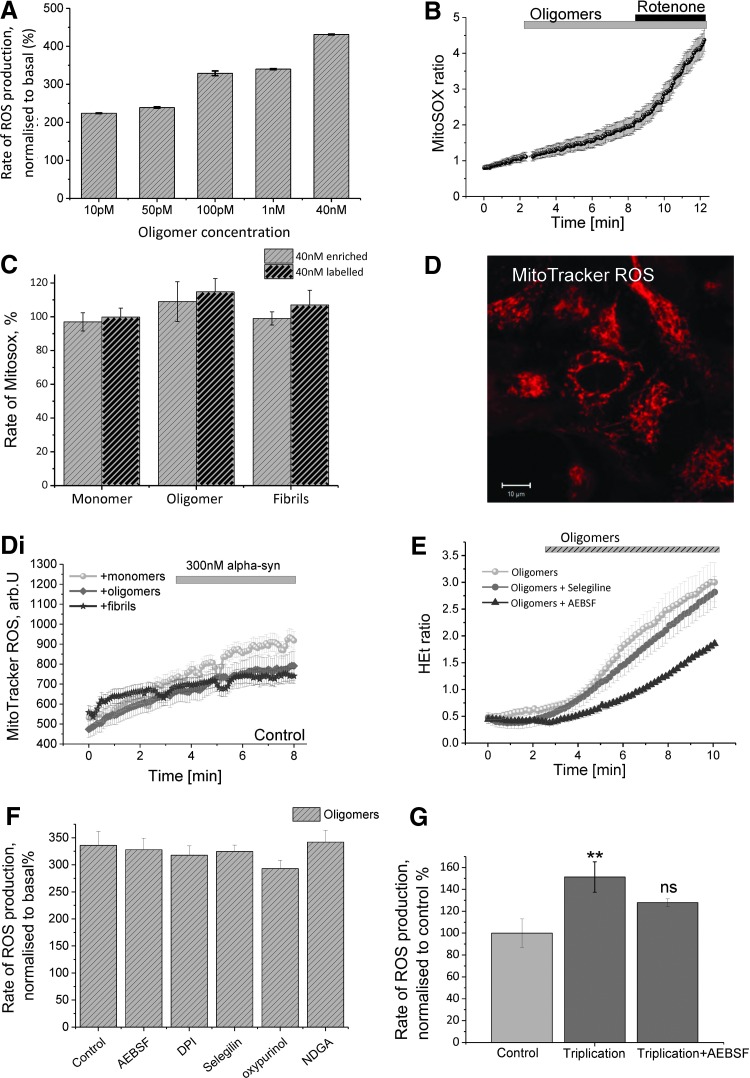
**α-S-induced ROS is independent of the major cellular ROS production systems. (A)** Unlabeled enriched oligomeric α-S was utilized to assess the dose-dependent effect of oligomers on ROS production. **(B)** Representative trace of mitochondrial ROS production (MitoSOX) in WT rat neurons upon oligomeric α-S exposure; 1 μ*M* rotenone induced a significant increase of MitoSOX fluorescence. **(C)** Equivalent levels of labeled and unlabeled enriched α-S species were assessed for their ability to induce mitochondrial ROS production in WT neurons using the mitochondrial ROS sensing dye, MitoSOX. **(D)** Representative image of iPSC-derived neurons labeled with MitoTracker red CM-H(2)X ROS. **(Di)** Representative traces of mitochondrial ROS production rate in the presence of monomeric, oligomeric, or fibrillar α-S in iPSC-derived neurons. **(E)** Representative traces showing the marginal decrease in ROS levels by AEBSF pretreatment of iPSC-derived neuron, **(F)** preincubation of WT neurons with inhibitors (DPI; Selegiline; Oxypurinol; NDGA) of the major cellular sources of ROS failed to block ROS production induced by the unlabeled enriched oligomeric species. **(G)** Quantification of ROS levels in control, α-S triplication, and AEBSF-treated α-S triplication neurons. *Bars* indicate the incubation period of either α-S species or rotenone. *n* = 3 experiments. Scale bar = 10 μm; error bars indicate SEM. **p < 0.01. AEBSF, 4-(2-aminoethyl)benzenesulfonyl fluoride hydrochloride; DPI, dibenziodolium chloride. To see this illustration in color, the reader is referred to the web version of this article at www.liebertpub.com/ars

To ensure that the ROS production observed in the neuronal cells is directly related to their exposure to oligomeric α-S species, we conducted a dose–response assay. Figure 3A demonstrates that exposing the neuronal cells to increasing concentrations of enriched oligomeric α-S, from 10 p*M* to 40 n*M* (mass concentration), resulted in a stepwise increase in cellular ROS production, indicating a direct correlation between the concentration of oligomeric protein and ROS production. It is worth noting that addition of oligomer solutions at a mass concentration of only 10 p*M* (which corresponds to ∼0.4 p*M* of molecules of oligomers), that is, concentration in terms of number of species (11), results in a significant increase in cytosolic ROS production, which shows the degree of specificity of this process and its relevance in the context of disease. All subsequent experiments using the enriched oligomers were conducted with a final concentration of 40 n*M* (mass concentration) α-S species unless otherwise stated.

### α-S-induced ROS is independent of the major cellular ROS production systems

The production of ROS in brain cells can be induced by enzymatic or nonenzymatic mechanisms (28). Mitochondria are considered to be one of the major sources of ROS production in resting cells. Application of 40 n*M* labeled or enriched monomeric, oligomeric, or fibrillar α-S to primary cocultures of neurons and astrocytes, loaded with the mitochondrial ROS indicator MitoSOX, had no significant effect (Fig. 3C), indicating that the oligomer-induced ROS production does not occur within the matrix of mitochondria. Subsequent application of 1 μ*M* rotenone induced a significant increase in the rate of MitoSOX ratio increase, confirming the ability of the MitoSOX probe fluorescence to detect mitochondrial ROS (Fig. 3B). We also used a further mitochondrial ROS indicator, MitoTracker red CM-H(2)X ROS, which resulted in good localization of the fluorescent indicator in the mitochondria of human iPSC-derived neurons (Fig. 3D). In agreement with MitoSOX data on primary neurons, monomeric, oligomeric, or fibrillar α-S did not change the rate of ROS production in the matrix of mitochondria of human iPSC-derived neurons (Fig. 3Di).

To investigate the source of oligomer-induced ROS production, we used a variety of inhibitors to block enzymes known to be responsible for intracellular ROS production. Inhibition of NADPH oxidase with 20 μ*M* of AEBSF or 0.5 μ*M* DPI had no effect on enriched oligomeric α-S-induced ROS production in neurons or astrocytes (Fig. 3F, *n* = 99 cells for AEBSF; and *n* = 104 cells for DPI—ROS levels were assessed for up to 30 min after α-S addition). Due to the lack of selectivity of the NADPH oxidase inhibitors available, we utilized two different ones in these experiments. Notably, DPI is a general flavoprotein inhibitor and therefore also induces inhibition of endothelial nitric oxide synthase, xanthine oxidase (XO), and certain proteins of the mitochondrial electron transport chain. AEBSF, in addition to its lower potency of NADPH oxidase inhibition, exerts inhibitory effects on serine proteases. However, taken together, these nonselective effects tend to overestimate, rather than underestimate, the role of NADPH oxidase (3).

Another important source of ROS in cells is the catabolism of monoamines *via* monoamineoxidases, (MAO) A and B (28). Selegiline is a selective and irreversible inhibitor of MAO-B at lower concentrations, but is also able to inhibit MAO-A at higher concentrations. Inhibition of this enzyme by 20 μ*M* selegiline also had no effect on the rate of ROS production induced by α-S in either neurons or astrocytes (Fig. 3F, *n* = 177 [*n* = 66 neurons and 111 astrocytes] and 3E shows representative traces). Inhibition of two further enzymes involved in cellular ROS production: XO (20 μ*M* oxipurinol, a noncompetitive irreversible inhibitor of XO, *n* = total 102 cells; 53 neurons and 49 astrocytes) and lipooxygenase (5 μ*M* nordihydroguai-aretic acid, *n* = total 81 cells; 32 neurons and *n* = 49 astrocytes) also had no effect on the oligomer α-S-induced ROS production (Fig. 3F). Notwithstanding the recognized nonspecific effects of certain inhibitors of cellular ROS production, these data suggest that the ROS production, generated upon neuronal exposure to oligomeric α-S, is independent of several of the known cellular ROS generation mechanisms.

Interestingly, treatment of the aged human triplication neurons (days 80–100) with AEBSF was able to partially reduce the high basal levels of ROS production (from 151% in triplication neurons ±13.89, *p* = 0.011 compared with control, to 127.9% in triplication neurons treated with AEBSF ±3.4, *n* = 13 neurons, *p* = 0.039), but could not completely block ROS production (Fig. 3G). These data strongly suggest that NADPH oxidase is not involved in the mechanism of ROS production induced specifically by oligomers, but may contribute to the production of damaging ROS species in human *SNCA* triplication neurons that have been exposed to high intracellular levels of α-S for prolonged periods of time. Under these circumstances, NADPH oxidase may be activated *via* several different cellular mechanisms, including alterations in calcium signaling (2, 37).

### α-S oligomers induce ROS *via* a redox metal ion-driven mechanism

It has been suggested that the toxicity of many aggregating peptides could be related to their ability to bind transition metal ions (9, 32). To determine if the α-S oligomers utilize their ability to bind metal ions to generate ROS in our experiments, we reassessed their ability to generate ROS in the presence of several metal chelators. For these experiments, we utilized the labeled oligomeric α-S characterized by single-molecule fluorescence.

**FIG. 4.**
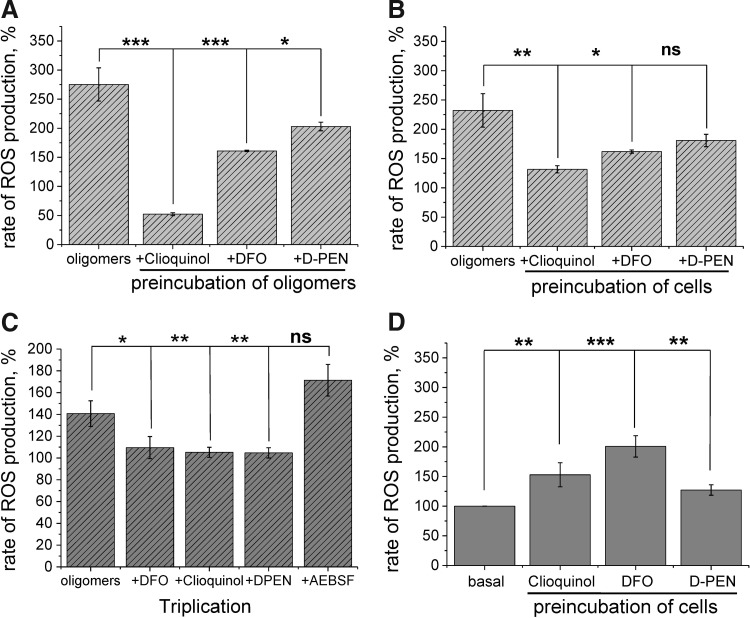
**α-S oligomers induce ROS** ***via*** **a redox metal ion-driven mechanism. (A)** Preincubation of the oligomers with different chelators, clioquinol, DFO, and D-PEN, for 10 min before addition to neurons resulted in a significant decrease in the oligomer-induced ROS signal. **(B)** Preincubation of cells with the same chelators for 15 min, followed by application of oligomers, also resulted in a significant reduction in the oligomer-induced ROS production. **(C)** Preincubation (15 min) of α-S triplication iPSC neurons cells with the chelators, followed by application of oligomers, resulted in significant decrease in oligomer-induced ROS production. Note that inhibitor of NADPH oxidase 20 μ*M* AEBSF had no effect on ROS production induced by oligomers in iPSC neurons. **(D)** Basal ROS production in cells preincubated with different chelators (clioquinol, DFO, and D-PEN) alone before the application of oligomers. *n* = 3 experiments. Error bars indicate SEM. *p < 0.05; **p < 0.05; ***p < 0.001. D-PEN, 1,2-diphenyl-1,2-ethylenediamine; DFO, desferrioxamine; iPSC, induced pluripotent stem cell.

We applied several metal chelators (2 μ*M* desferrioxamine [DFO]—iron chelator, 50 μ*M* D-pencillamine—copper chelator, and 50 μ*M* clioquinol [CLQ]—highly lipophilic copper and zinc chelator with moderate affinity for iron binding) directly to the oligomers for 10 min before application of the mixture to cells. In the presence of all the chelators, the ability of the oligomers to induce ROS was significantly reduced (52.2% ± 2.6% for CLQ, *n* = 62, *p* < 0.0001; 160.9% ± 1.3% for DFO, *n* = 58, *p* = 0.0006; and 202.8 ± 7.4, *n* = 55, *p* = 0.0351 for D-PEN compared with oligomers alone, 275.3% ± 28.6%, *n* = 75; Fig. 4A). Next, we preincubated cells with the same chelators for 10 min before the application of oligomers. Treatment of cells with the chelators alone (before application of oligomers) did produce a variable increase in the basal ROS (for CLQ 152% ± 20.3%, *n* = 54, *p* = 0.003, for DFO 200.7% ± 18.1%; *n* = 58; *p* < 0.001, and for D-PEN 127.2% ± 8.9%; *n* = 87; *p* = 0.0023; Fig. 4D). However, despite these changes in the basal ROS production, pretreatment with the chelators significantly prevented ROS production on application of oligomers (131.59% ± 6.2% for CLQ, *n* = 67, *p* = 0.0028; 162.0% ± 2.8% for DFO, *n* = 74, *p* = 0.0275; and not statistically significant for D-PEN-180.8% ±10.6%, *n* = 54, *p* = 0.1752; compared with oligomers without pretreatment with chelators 232.5% ± 28.6%, *n* = 87; Fig. 4B).

Importantly, the ability of metal chelator to reduce the oligomer-induced ROS production was also tested on iPSC-derived control and *SNCA* triplication neurons. For these experiments, neurons were used at day 50 in culture so that the basal levels of ROS were not significantly different. Cells were preincubated for 10 min with vehicle, CLQ, DFO, D-PEN, or AEBSF, and then 300 n*M* total α-S (3 n*M* oligomers) was applied to the chamber. As confirmed in the aged human neurons, the application of the oligomeric α-S to young control or *SNCA* triplicated iPS neurons induced an increase in the rate of ROS production. Oligomer-induced ROS production in the iPSC-derived *SNCA* triplication neurons (140% ± 11.7%; *n* = 67 cells) could be reduced to control levels by CLQ (105% ± 4.6%; *n* = 54 cells; *p* = 0.0014), D-PEN (104% ± 4.7%; *n* = 87; *p* = 0.007) or DFO (109% ± 10.1%; *n* = 58 cells; *p* = 0.019), but not by the inhibitor of NADPH oxidase AEBSF (171% ± 14.5%; *p* = 0.111; *n* = 3 experiments, Fig. 4C).

These combined data suggest that oligomeric α-S is able to produce significant levels of superoxide radicals by a redox metal-driven mechanism in neuronal model systems. More specifically, copper and iron can be utilized by the oligomeric species to produce ROS. Chelation of the metal ions before application to cells and chelation of metal ions within cells are both effective at preventing the oligomer-induced ROS production.

### Exposure of neuronal cultures to oligomeric α-S reduces endogenous glutathione levels and triggers caspase-3/7 activation

ROS is normally produced in healthy cells during metabolic processes and known to have an important role as signaling molecules (25, 51). Oxidative stress is defined as a misbalance in cell redox reactions, resulting in the increase of ROS and decreased antioxidant defense systems. To identify whether α-S-induced ROS production can induce oxidative stress, we measured the level of the major antioxidant in brain—glutathione (GSH).

**FIG. 5.**
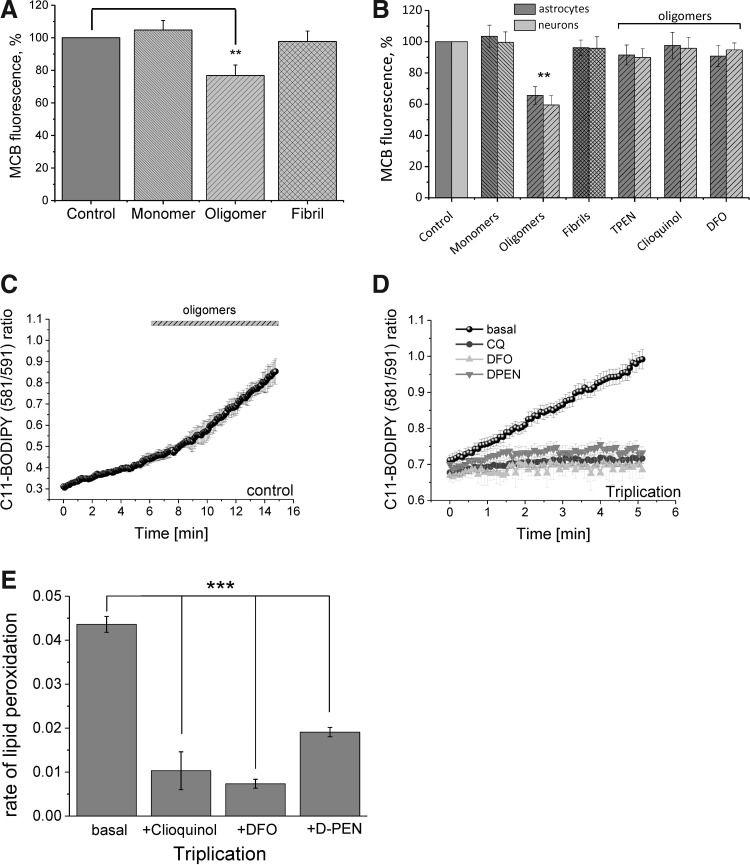
**Exposure of neuronal cultures to oligomeric α-S reduces endogenous GSH levels and increases lipid peroxidation. (A)** Reduced GSH levels of WT rat neurons after incubation with the different α-S species for 48 h; the levels of reduced GSH were assayed using MCB. **(B)** Treatment of neurons and astrocytes with the metal chelators, TPEN, DFO, or clioquinol, prevents the decrease in endogenous GSH levels caused by the oligomer-induced ROS production. **(C)** Control and α-S triplication cells were loaded with C11-Bodipy and lipid oxidation was recorded. Representative trace of oligomeric-α-S-induced lipid peroxidation in control iPSC neurons. **(D)** Representative traces of lipid peroxidation in α-S triplication cells preincubated with D-PEN, DFO, clioquinol, or solvent. **(E)** Quantification of lipid peroxidation rate in α-S triplication iPSC neurons preincubated with chelators or solvent. *Bar* indicates the incubation period of oligomers. *n* = 3 experiments. Error bars indicate SEM. **p < 0.01; ***p < 0.001. GSH, glutathione; MCB, monochlorobimane; TPEN, N,N,N′,N′-tetrakis(2-pyridylmethyl)ethylenediamine

We measured the cellular levels of GSH using monochlorobimane (MCB) after 24 h of exposure to different forms of α-S. Enriched oligomeric α-S (at a final concentration of 40 n*M*) significantly decreased the levels of reduced GSH in neurons and astrocytes (to 76.8% of control in neurons; *n* = 4 experiments; *p* < 0.001; and to 84.7% in astrocytes, *n* = 4; *p* < 0.001; Fig. 5A), while the monomeric form had no effect on the MCB fluorescence (*n* = 4). Importantly, although fibrils produced an increase in ROS production, the levels of ROS were insufficient to decrease the levels of reduced GSH and therefore to induce oxidative stress in the neuronal cells (*n* = 4 experiments; Fig. 5A). Given our observations that metal chelators were able to inhibit oligomer-induced ROS production, we further assessed the ability of these chelators to rescue the reduction in endogenous levels of reduced GSH. Strikingly, pretreatment of cells with any of these chelators for 15 min before oligomeric exposure was sufficient to prevent the reduction in GSH levels in both neurons and astrocytes (Fig. 5B).

Activation of lipid peroxidation is another indication of oxidative stress occurring within cells. Previously, we showed that oligomeric α-S increased the rate of lipid peroxidation in primary neurons and astrocytes (4). In agreement with these findings, we found that oligomeric α-S also stimulated lipid peroxidation in control human iPSC-derived neurons (Fig. 5C), as confirmed by an increase in the C11-BODIPY (581/591) fluorescence ratio. We further studied the basal rate of lipid peroxidation in the triplication neurons using the increase in fluorescence ratio of C11-BODIPY in the first 5 min following preincubation of cells with different chelators. We have demonstrated that the oligomer-induced lipid peroxidation in human iPSC-derived *SNCA* triplication neurons could be significantly reduced by treatment of these cells with CLQ (oligomer-treated triplication cell value 0.0436 ± 0.002, *n* = 82 reduced to 0.01031 ± 0.0043, *n* = 75), DFO (reduced from 0.0436 ± 0.002 to 0.00736 ± 0.001, *n* = 84) and D-PEN (reduced from 0.0436 ± 0.002 to 0.0191 ± 0.001, *n* = 64), with *p*-values for each chelator *versus* control *p* < 0.0001 (Fig. 5D, E).

**FIG. 6.**
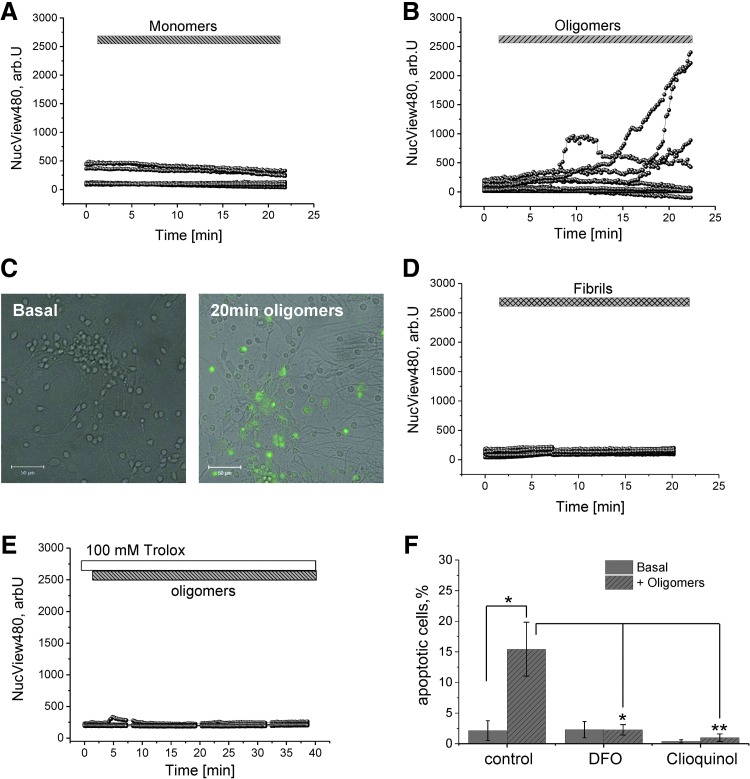
**α-S oligomers trigger oxidative stress-induced caspase-3 activation in neurons.** WT rat midbrain neurons were loaded with NucView488 caspase-3 substrate, which fluoresces green upon caspase-3/7 activation. Neurons were exposed to **(A)** monomeric, **(B)** oligomeric, and **(D)** fibrillar α-S, respectively. **(C)** Representative image of basal and oligomeric α-S-induced caspase-3 activation. **(E)** Representative traces demonstrating the inhibition of oligomeric-induced caspase-3 activation in cells preincubated with 100 μ*M* Trolox. **(F)** Quantification of apoptosis (caspase-3 activation) in untreated, DFO, or clioquinol-treated WT rat neurons exposed to PBS or oligomeric α-S. *Bars* indicate the incubation period of either α-S species or Trolox. Scale bar = 50 μm; *n* = 4 experiments; error bars indicate SEM. *p < 0.05; **p < 0.01. To see this illustration in color, the reader is referred to the web version of this article at www.liebertpub.com/ars

To investigate if the levels of ROS production and oxidative stress induced by oligomeric α-S could directly contribute to cell death, we examined the effect of the different forms of α-S on caspase-3/7 activation, one of the major enzymes involved in the cell death cascade. We used the NucView 488 caspase-3 substrate, which allows the visualization of caspase-3/7 activation in real time. The increase in the emitted 488 nm fluorescence in the nuclear region of cells reflects the cleavage of the caspase-3 substrate (NucView) by activated caspase-3/7 and the migration of the released fluorescent dye to the nucleus. An increase in fluorescence as indicated by the representative traces of individual cells in Figure 6 demonstrates an increase in caspase-3/7 activation and cleavage of the fluorescent substrate. Application of oligomeric α-S induced a rapid activation of caspase-3/7 in neurons (14.5% in 20 min of exposure; *n* = 4 experiments) and astrocytes (11.6% in 20 min of exposure; *n* = 4 experiments; Fig. 6B, C), whereas monomeric or fibrillar α-S failed to activate caspase-3 (Fig. 6A, D).

To investigate whether α-S-induced caspase-3/7 activation is directly mediated by ROS accumulation, we assessed neuronal death by caspase-3/7 activation by treating cell cultures with trolox (a water-soluble derivative of vitamin E), an antioxidant agent commonly used to reduce oxidative stress in cells. Pretreatment of the cells with trolox, before the application of oligomeric α-S, prevented caspase-3/7 activation by oligomeric α-S (Fig. 6E; *n* = 3 experiments). This suggests that the oligomeric α-S toxicity is driven mainly by the generation and accumulation of ROS inside neuronal cells. Given that our previous data suggested that treatment with the metal chelators can alleviate both the aberrant ROS production and reduction in reduced endogenous GSH levels, we assessed whether treatment with the metal chelators could also prevent caspase-3/7 activation. For these experiments, single snap images were taken from five randomly selected fields of view at baseline and at 30 min following application of oligomers. Untreated cells showed a low level of basal caspase-3/7 activation (2.1% ± 1.4%, *n* = 315 cells; Fig. 6F), which was significantly increased upon addition of the α-S oligomers (15.4% ± 4.4%; *n* = 316 cells; *p* = 0.017; Fig. 6F). In contrast, pretreatment of the cells with either DFO (2.2% ± 0.86%; *n* = 598 cells; *p* = 0.014; Fig. 6F) or CLQ (0.97% ± 0.67%; *n* = 368 cells; *p* = 0.008; Fig. 6F) was sufficient to protect against caspase-3 activation.

## Discussion

α-S overexpression and misfolding are critical triggers in the pathogenesis of PD. The interaction between α-S and oxidative stress in PD models is considered to be complex and bidirectional such that oxidative stress may promote the aggregation of α-S, while at the same time the misfolding of α-S is associated with increased ROS production. In this study, we focused on the latter effect, that is, the ability of α-S to intrinsically generate ROS, and the mechanism by which this is achieved.

Previous studies have reported that overexpression of wild-type or mutant α-S *in vitro* increases sensitivity to dopamine toxicity (33, 48). Overexpression of wild-type and mutant α-S can also induce ROS in neuroblastoma cells and enhance their vulnerability to dopamine (33). Overexpression of wild-type or mutant α-S *in vivo* models also results in increased sensitivity to toxins inducing oxidative stress, including MPTP and 6-OHDA (42), while mice lacking α-S are resistant to MPTP and mitochondrial toxins (15). It has been previously reported that application of recombinant aggregated α-S (in the form of preformed fibrils) induces an increase in cytosolic and mitochondrial ROS production (26). Concluding that both expression and aggregation of α-S may be fundamental to its pathogenesis, we utilized models and tools to study both the effect of long-term increase in endogenous α-S in human cells and the structure-specific effects of α-S on mechanisms of oxidative stress.

Patients bearing a triplication of the *SNCA* locus have early onset, autosomal dominant PD associated with α-S inclusions pathologically. iPSC-derived neurons from a patient bearing the *SNCA* triplication recapitulate the doubling of α-S protein found in patients and therefore provide an ideal model for studying PD pathogenesis and its relationship with α-S pathology (20). We report that human neurons with higher levels of endogenous α-S for long periods of time in culture exhibit high levels of ROS in the forms of superoxide and H_2_O_2_. Thus, we confirm that increased expression of intracellular α-S does indeed result in increased generation of ROS at basal levels in a familial model of PD.

It is not yet clear whether the α-S-induced ROS production is related to the level of expression of α-S or whether it is an effect of protein misfolding and aggregation. It is technically challenging to assess the different structural species of endogenous α-S that exist within cells at high molecular resolution. We therefore produced recombinant α-S and generated samples of different structural forms of the protein that correspond to different degrees of aggregation in the protein. Of note, we employed two different types of aggregation methods to generate the oligomeric α-S preparation. In the first method, the protein was fluorescently labeled and thus the oligomeric fraction of the preparation could be characterized using singe-molecule fluorescence techniques such as smFRET (14). Utilizing this method of oligomer generation, we confirmed that the beta-sheet-rich compact oligomers were the most highly damaging to human neurons due to their unique ability to generate ROS. However, this particular oligomer preparation consists of ∼1% oligomer and therefore the presence of 99% monomeric α-S needs to be considered in all experiments.

In this study, we also therefore utilized a new method of oligomer generation that was able to form unlabeled preparations that were highly enriched in oligomers (90%).The precise molecular structure of the intermediate species, the oligomers, from this protocol was fully characterized using biophysical techniques and found to resemble the most highly compact and stable species that constitute the previous oligomer preparations. Thus, we were able to compare directly the effects of pure samples of monomeric, soluble oligomeric, and fibrillar α-S on wild-type neurons. We have previously shown that all the α-S species and conformations analyzed in this study are rapidly taken up by both neurons and astrocytes (within 5–10 min), without significant variations between protein species (14), which allows us to directly correlate cellular readouts with the amounts of different protein species added to the cells. Importantly, we note that both oligomeric preparations were consistent in their ability to generate high levels of ROS in primary neurons and demonstrated the same mechanism of ROS production. Some differences between the potency in ROS production were observed between the two preparations, and this may be due to inherent structural differences between the oligomers and also to the difficulty in using equivalent oligomer concentrations for cellular experiments.

In this study, we demonstrate that α-S in its oligomeric state is a highly potent inducer of superoxide production, able to generate aberrant cytosolic ROS production even at low picomolar concentrations of species. These effects were demonstrated using different oligomer preparations, different ROS assays in two different cell models (primary neuronal cocultures and human iPSC-derived cortical neurons). Thus, we show that the misfolding of α-S into oligomers with particular structural characteristics is critical to confer the toxic properties that can induce ROS production (while other structural protein species are significantly less efficient in inducing ROS production) (11). This process, furthermore, is also associated with depletion of GSH, reflecting an inability of the antioxidant defenses to manage the high levels of ROS produced (and thus leading to the state of oxidative stress). High levels of oxidative stress were further highlighted by the demonstration of elevated lipid peroxidation induced by oligomeric α-S. Ultimately, the α-S-induced oxidative stress is sufficient to result in apoptosis and cell death. All together, these results suggest that the formation and accumulation of certain structural groups of soluble oligomeric forms of α-S are important events in the pathogenesis of PD.

Several mechanisms have been proposed to account for α-S-induced ROS production. These include (i) the interaction of α-S protofibrils with vesicular membranes (52), resulting in disruption of membrane integrity and leakage of catecholamine and oxidized metabolites into the cytosol (41); (ii) α-S-induced clustering of the dopamine transporter, resulting in greater uptake of dopamine and higher cytosolic concentrations of dopamine (39); (iii) binding of α-S to the inner mitochondrial membrane with impairment of complex 1 activity and generation of ROS (12, 19); and (iv) activation of cytosolic NADPH oxidase by α-S aggregates leading to ROS production (26). We tested a range of cellular processes that generate ROS and we found that the α-S oligomer-induced ROS production was remarkably independent of several of these processes, specifically mitochondrial matrix ROS generation, catecholamine metabolism, and cytosolic NADPH oxidase activity. We therefore concluded that the oligomers were potentially able to generate ROS through a nonenzymatic process that may be related to the specific physicochemical and structural properties of certain oligomeric species.

Interestingly, incubation of the oligomers themselves with metal chelators (of copper and iron) before application to cells abolished their ability to generate ROS. This suggested that the oligomers may interact with metal ions and that these metal ions may be responsible for the ability of the oligomers to induce ROS. Preincubation of the cells with chelators before application of oligomers was also able to prevent the oligomer-induced ROS generation and, furthermore, had the effect of reversing the depletion of GSH and reversing the apoptosis induced by oligomers. Thus, metal chelation within cells and with oligomers was a potent strategy to prevent oligomer-induced oxidative stress and oligomer-induced cell death.

The nature of the synergistic interaction between metal ions and oligomers remains unresolved, although we may speculate whether it represents a direct or indirect interaction. It is well established that α-S can interact with metal ions in a nonspecific manner or with low-affinity or high-affinity binding sites. These metal ion interactions modify the structure of α-S and alter the propensity of it to aggregate into fibrils (45). Monomeric α-S shows the highest affinity, in the nanomolar range, for copper (6) and this metal is able to undergo reversible redox cycling when bound to the protein (17). α-S also binds iron and this was originally thought to be a low-affinity interaction, although recent studies have suggested that it can bind iron in the low micromolar range (16). Purified recombinant α-S has been reported to be able to reduce Fe^3+^ to Fe^2+^, opening the possibility that α-S could act as a cellular iron-reducing protein or ferrireductase (7). Interestingly, monomeric α-S, like other proteins associated with neurodegeneration such as the Aβ peptide and the prion protein, has been reported to be able to generate H_2_O_2_ and hydroxyl radicals *in vitro* in the presence of small amounts of Fe^2+^, suggesting further that α-S can directly generate ROS in a metal-dependent manner (47, 50). High cellular levels of copper or iron result in cellular toxicity, and the toxicity has been attributed to the redox activity of these metal ions that can lead to the production of ROS through Haber–Weiss or Fenton reactions (40).

It is of significant relevance that dysregulation of iron homeostasis with resultant iron overload is well recognized in PD and in several other synucleinopathies. Postmortem analysis of PD brains reveals significantly higher levels of iron in the substantia nigra (23) and within Lewy bodies (10). Increased iron has also been demonstrated in the substantia nigra of patients with PD using transcranial sonography and T2*-weighted MRI. A putative iron regulatory element has been reported in the 5′ UTR of the mRNA encoding α-S, suggesting that the regulation of the α-S protein production may occur in response to changes in iron or redox events (27). Of note, modulation of iron using chelator therapy has reduced neuronal loss in the MPTP or 6-OHDA PD toxin models (34). Indeed, desferiprone, a membrane-permeant bidentate chelator that scavenges labile iron and confers protection against iron-induced oxidative stress, has been shown to prevent iron deposition in the substantia nigra and encouragingly slow disease progression in early phase clinical trials (21). Our data suggest that the toxic oligomeric forms of α-S are able to interact with metal ions such as iron and copper and induce the production of significant levels of ROS within cells, leading to metal-dependent oxidant stress and cell toxicity. This work provides a valid basis for the strategy of metal chelation as a neuroprotective therapy in PD.

In summary, we have shown that production of ROS and subsequent oxidative stress represents a major pathogenic pathway in alpha-synucleinopathies. α-S in physiological concentrations and in its monomeric state does not generate production of ROS or toxicity. However, once the process of aggregation begins, very small concentrations of toxic oligomeric species formed at the early stages of the self-assembly process are sufficient to induce massive ROS production, oxidative stress, and apoptosis. The mechanism by which the toxic oligomer generates ROS is directly associated with redox metal ions and we show that it can be prevented by the chelation of free iron or copper. This work unravels the interaction between protein misfolding, metal homeostasis, and oxidative stress and highlights the rational basis for a novel therapeutic strategy in PD.

## Materials and Methods

### Preparation of the different α-S species samples

Human α-S was overexpressed and purified as a monomeric fraction from *Escherichia coli* as described previously (31). Fibrils were prepared by incubating monomeric α-S at 70 μ*M* (1 mg/ml) in phosphate-buffered saline (PBS) buffer pH 7.4 (0.1 *M* ionic strength; 0.01% NaN_3_ was added to prevent bacterial growth during aggregation) at 37°C under constant agitation (200 rpm) for 4–6 days. After this time, the samples were centrifuged (10 min at 13.200 rpm) and the fibrillar pellet was washed twice with PBS before being resuspended into the appropriate volume of PBS to give ∼100 μ*M* of fibrillar sample. Samples of α-S oligomers were prepared by incubating monomeric protein at ∼800 μ*M* (12 mg/ml) in PBS at 37°C without agitation for 18–22 h. To get this high concentration of protein, a solution of monomeric α-S in miliQ water was lyophilized and typically 6 mg of lyophilized α-S was resuspended in 500 μl of PBS buffer. The resulting solution was passed through a filter device with a 0.22 μ*M* cutoff to remove any particles of dust and/or large protein aggregates that could have been formed during the lyophilization process. After incubating this solution at 37°C, without agitation, for 18–22 h, most of the protein remains monomeric, but a fraction of ∼5% of the protein is in the form of aggregates. These aggregates are still soluble after ultracentrifugation at 90,000 rpm for 1 h. To isolate these aggregates from the monomeric protein, a series of filtering devices with a 100 kDa cutoff were used so that the monomeric protein passed through the membrane, while oligomeric species bigger than 100 kDa remained at the top of the filter.

Fluorescently labeled oligomers were generated by the same procedure by mixing a sample of 400 μ*M* of Alexa Fluor-488 (AF488)-labeled protein with one of 400 μ*M* Alexa Fluor-647 (AF647)-labeled protein to give a solution with a total protein concentration of 800 μ*M* in PBS. The A90C mutant variant of α-S was expressed in, and purified from, *E. coli* as described previously (31) and labeled with either maleimide-modified AF488 or AF647 dyes (Invitrogen) *via* the cysteine thiol moiety (at labeling efficiencies of 98% ± 3% for both cases as estimated by mass spectrometry). The labeled protein was purified from the excess of free dye by a P10 desalting column with Sephadex G25 matrix (GE Healthcare), divided into aliquots, flash-frozen, and lyophilized; the lyophilized protein was stored at −20°C.

For the single-molecule FRET analysis of the fluorescently labeled oligomers, a 2 μl aliquot was diluted 10^5^-fold by serial dilution with 0.022 μm filtered Tris 25 m*M*, pH 7.4, 0.1 *M* NaCl. Glass slides were incubated for 1 h with bovine serum albumin (BSA) at 1 mg/ml to prevent α-S species from adsorbing to the surface, as we have recently shown (14). Immediately after removal of the BSA solution, 500 μl of diluted sample was placed on the slide for analysis.

### Human control and triplication iPSC culture and differentiation

iPSCs were maintained on irradiated SNL feeders (available from European Collection of Cell Cultures, ECACC) and supplemented with hESC medium (*Knockout DMEM*, 20% KSR, 2 m*M* L-glutamine, 1× nonessential amino acids, 50 μ*M* 2-mercaptoethanol, 50 U/ml penicillin, 50 μg/ml streptomycin [all from Invitrogen], and 20 ng/ml FGF2 [Peprotech]). iPSCs were then subjected to a dual SMAD inhibition protocol for neuronal differentiation. To commence neuronal differentiation, iPSC cultures were dissociated with Accutase (Invitrogen) to generate single-cell suspensions, then differentially plated on gelatine-coated plasticware for 1 h in hESC medium in the presence of ROCK inhibitor (Y27632; Ascent) to remove SNL feeders. Nonattached cells were resuspended in MEF-CM (R&D Systems) with 10 ng/ml FGF2 (Peptrotech) and plated on hESC-grade Matrigel (BD) at a density of 50,000 cells/cm^2^ and allowed to propagate in self-renewal conditions for 72 h or until 90% confluent, whereupon media were changed to KSR medium containing 100 n*M* LDN-193189 (Stemgent) and 10 μ*M* SB431542 (Tocris) added daily. After 5 days, KSR medium containing 100 n*M* LDN-193189 was cross-tapered with N2B27 medium (Stem Cells) containing 100 n*M* LDN-193189 over 7 days (75% KSR and 25% N2B27 first day, 50% of each third day, then 25% KSR and 75% N2B27 fifth day). At day 11, cells were dissociated with Accutase (Invitrogen) and replated at a 1:2 ratio on plasticware coated with 5 μg/ml laminin and 5 μg/ml fibronectin (both from Sigma) in N2B27 media supplemented with 20 ng/ml BDNF and 10 ng/ml GDNF (both from Peprotech). Thereafter, media were half changed on alternate days and cells were maintained in culture for ∼80 days before experiments were carried out.

The generation of cortical neurons using this protocol was tested using immunocytochemistry for neuronal markers. Control and *SNCA* triplication iPSC-derived neurons growing on iBidi slides were fixed in 4% paraformaldehyde for 20 min at room temperature, washed in PBS once, and permeabilized with 0.2% Triton-100 for 20 min at room temperature. The cells were then blocked in PBS containing 5% BSA for 1 h at room temperature, followed by overnight incubation with primary antibodies overnight at 4°C. Mouse monoclonal anti-beta-III-tubulin antibody (Abcam ab7751; 1:500 dilution) was used for labeling the neuronal population. Alexa Fluor-488-conjugated anti-mouse secondary antibody was used for visualization. Cells were imaged using a Zeiss 710 CLSM confocal system.

Differentiation into cortical neurons was further validated by testing the calcium response to physiological neuronal stimuli. Control and *SNCA* triplication iPSC-derived neuronal cultures were loaded for 30 min at room temperature with 5 μ*M* fura-2 AM and 0.005% Pluronic in an HEPES-buffered salt solution (HBSS) comprising (m*M*) 156 NaCl, 3 KCl, 2MgSO_4_, 1.25 KH_2_PO_4_, 2 CaCl_2_, 10 glucose, and 10 HEPES, pH adjusted to 7.35 with NaOH; 100 μ*M* ATP was applied to cells to stimulate a calcium signal in astrocytes, and this was followed by 5 μ*M* glutamate to stimulate a calcium signal in neurons. Fluorescence measurements were obtained on an epifluorescence inverted microscope equipped with a 20**×** fluorite objective. [Ca^2+^]_c_ was monitored in single cells using excitation light provided by a Xenon arc lamp, the beam passing the monochromator at 340, 360, and 380 nm (Cairn Research). Emitted fluorescence light was reflected through a 515 nm long-pass filter to a cooled CCD camera (Retiga; QImaging) and digitized to 12-bit resolution. All imaging data were collected and analyzed using software from Andor.

### Primary neuronal culture preparation

Mixed neuronal midbrain cultures were prepared from Sprague-Dawley rat pups 3 days postpartum (UCL breeding colony). Subjects were culled *via* schedule-1 decapitation and the midbrain was dissected into ice-cold HEPES-buffered salt solution (Ca^2+^, Mg^2+^-free; Gibco-Invitrogen). The tissue was minced and trypsinized (0.25% for 15 min at 37°C), triturated, and plated on poly-D-lysine-coated 22-mm coverslips and cultured in neurobasal A medium (Gibco-Invitrogen) supplemented with B-27 (Gibco-Invitrogen) and 2 m*M* glutamax. Cultures were maintained at 37°C in a humidified atmosphere of 5% CO_2_ and 95% air, fed once a week, and maintained for a minimum of 14 days before experimental use. Neurons were easily distinguishable from glia: they appeared phase bright, had smooth rounded somata and distinct processes, and lay just above the focal plane of the glial layer. Cells were used at 14–16 days *in vitro*. Animal husbandry and experimental procedures were performed in full compliance with the United Kingdom Animal (Scientific Procedures) Act of 1986.

### ROS assessments

Fluorescence measurements were obtained on an epifluorescence inverted microscope equipped with a 20× fluorite objective. For HEt and MitoSOX measurements, ratios of the oxidized to reduced forms of the dye were measured: excitation at 540 nm and emission recorded above 560 nm were used to quantify the oxidized form (ethidium), whereas excitation at 360 nm and emission collected from 405 to 470 were used for the reduced form (hydroethidium). All data reported in this study were obtained from at least five coverslips and two to three different cell and sample preparations.

For measurement of mitochondrial ROS production, cells were preincubated with MitoSOX (5 μ*M*; Molecular Probes) or MitoTracker Red CM-H(2)XROS for 10 min at room temperature. For measurement of cytosolic ROS production, HEt (2 μ*M*) was present in the solution during the experiment. No preincubation (loading) was used for HEt to limit the intracellular accumulation of oxidized products. MitoTracker Red CM-H(2)XROS measurements were produced using 560 nm excitation and emission above 580 nm.

For the intracellular H_2_O_2_ assessments using the Hyper constructs, primary rat neurons and human iPSCs were transfected with the Hyper-3 construct or the Hyper-CS control pH probe using Effectene according to the manufacturers' instructions (QIAGEN). Importantly, due to the toxicity of the Effectene reagent in combination with Neurobasal A media, cells were exposed to the transfection complexes for an hour before the media were replaced with normal Neurobasal A growth medium. Cells were then left to express the constructs for 48 h before experiments were performed. Hyper-CS/3 were excited at 488 and 405 nm and emission was set to 510–540 nm and values were expressed as 488/405 ratio (5, 44).

The rate of lipid peroxidation was measured using confocal microscopy. Confocal images were obtained with a Zeiss 710 LSM with an integrated META detection system. To assess lipid peroxidation, C11-BODIPY (581/591, 2 μ*M*; Molecular probes) was excited using the 488 and 543 nm laser line and fluorescence measured using a band-pass filter from 505 to 550 nm and 560 nm long-pass filter (40× objective). Illumination intensity was kept to a minimum (at 0.1–0.2% of laser output) to avoid phototoxicity and the pinhole set to give an optical slice of ∼2 μm. Addition of a bright-field image allowed separation between neurons and glia that are visibly different and are situated on different focal planes. Data were acquired and analyzed using ZEN2009 software.

### GSH assessments

Neuronal cultures were incubated with 50 μ*M* MCB (Molecular Probes, Invitrogen) for 40 min in HEPES-buffered salt solution before imaging (1, 36). Cells were then washed with HEPES-buffered salt solution and images of the fluorescence of the MCB-GSH were acquired using a Zeiss UV-vis 510 CLSM with excitation at 351 nm and emission at 435–485 nm.

### Caspase-3 assays

Neuronal cultures were incubated with 5 μl of NucView 488 reagent (Biotium) per ml of culture media for 20 min at 37°C, 5% CO_2_. Cells were then imaged using a Zeiss 710 LSM confocal microscope equipped with an META detection system and an ×40 oil immersion objective.

For measurements of caspase-3/7 activation, cells were loaded for 15 min at room temperature with 10 μ*M* NucView 488 caspase-3 substrate (Biotium) in HBSS. NucView 488 is a novel class of enzyme substrates for real-time detection of caspase-3/7 activity in live cells. The substrate can rapidly cross the cell membrane to enter the cell cytoplasm, where it is cleaved by caspase-3/7 to release the high-affinity DNA dye. The released DNA dye migrates to the cell nucleus to stain the nucleus bright green.

Confocal images were obtained using a Zeiss 710 confocal laser scanning microscope and a 40× oil immersion objective. The 488 nm argon laser was used to excite NucView 488 fluorescence, which was measured using a band-pass filter from 510 and 560 nm.

## Abbreviations Used

6-OHDA: 6-hydroxydopamine
AEBSF: 4-(2-aminoethyl)benzenesulfonyl fluoride hydrochloride
BSA: bovine serum albumin
CLQ: clioquinol
DFO: desferrioxamine
DHE: dihydroethidium
D-PEN: 1,2-diphenyl-1,2-ethylenediamine
DPI: dibenziodolium chloride
GSH: glutathione
H_2_O_2_: hydrogen peroxide
HBSS: HEPES-buffered salt solution
hESC: human embryonic stem cells
HEt: dihydroethidium
iPSC: induced pluripotent stem cell
KSR: knockout serum replacement
MAO: monoamineoxidase
MCB: monochlorobimane
MPTP: mitochondrial permeability transition pore
NDGA: nordihydroguaiaretic acid
PBS: phosphate-buffered saline
PD: Parkinson's disease
ROS: reactive oxygen species
*SNCA*: alpha-synuclein
SNL: immortalized cell line derived from mouse fibroblast cells
TPEN: N,N,N′,N′-tetrakis(2-pyridylmethyl)ethylenediamine
XO: xanthine oxidase
WT: wild-type
α-S: alpha-synuclein
